# Stereotactic body radiation therapy for melanoma and renal cell carcinoma: impact of single fraction equivalent dose on local control

**DOI:** 10.1186/1748-717X-6-34

**Published:** 2011-04-08

**Authors:** Michelle A Stinauer, Brian D Kavanagh, Tracey E Schefter, Rene Gonzalez, Thomas Flaig, Karl Lewis, William Robinson, Mark Chidel, Michael Glode, David Raben

**Affiliations:** 1 University of Colorado Denver, School of Medicine, Aurora, Colorado, USA; 2 Exempla St. Joseph Hospital, Denver, Colorado, USA

## Abstract

**Background:**

Melanoma and renal cell carcinoma (RCC) are traditionally considered less radioresponsive than other histologies. Whereas stereotactic body radiation therapy (SBRT) involves radiation dose intensification via escalation, we hypothesize SBRT might result in similar high local control rates as previously published on metastases of varying histologies.

**Methods:**

The records of patients with metastatic melanoma (n = 17 patients, 28 lesions) or RCC (n = 13 patients, 25 lesions) treated with SBRT were reviewed. Local control (LC) was defined pathologically by negative biopsy or radiographically by lack of tumor enlargement on CT or stable/declining standardized uptake value (SUV) on PET scan. The SBRT dose regimen was converted to the single fraction equivalent dose (SFED) to characterize the dose-control relationship using a logistic tumor control probability (TCP) model. Additionally, the kinetics of decline in maximum SUV (SUV_max_) were analyzed.

**Results:**

The SBRT regimen was 40-50 Gy/5 fractions (n = 23) or 42-60 Gy/3 fractions (n = 30) delivered to lung (n = 39), liver (n = 11) and bone (n = 3) metastases. Median follow-up for patients alive at the time of analysis was 28.0 months (range, 4-68). The actuarial LC was 88% at 18 months. On univariate analysis, higher dose per fraction (p < 0.01) and higher SFED (p = 0.06) were correlated with better LC, as was the biologic effective dose (BED, p < 0.05). The actuarial rate of LC at 24 months was 100% for SFED ≥45 Gy v 54% for SFED <45 Gy. TCP modeling indicated that to achieve ≥90% 2 yr LC in a 3 fraction regimen, a prescription dose of at least 48 Gy is required. In 9 patients followed with PET scans, the mean pre-SBRT SUV_max _was 7.9 and declined with an estimated half-life of 3.8 months to a post-treatment plateau of approximately 3.

**Conclusions:**

An aggressive SBRT regimen with SFED ≥ 45 Gy is effective for controlling metastatic melanoma and RCC. The SFED metric appeared to be as robust as the BED in characterizing dose-response, though additional studies are needed. The LC rates achieved are comparable to those obtained with SBRT for other histologies, suggesting a dominant mechanism of in vivo tumor ablation that overrides intrinsic differences in cellular radiosensitivity between histologic subtypes.

## Background

For at least three decades, renal cell carcinoma (RCC) and melanoma have been considered to be relatively "radioresistant" tumors. In the case of RCC, this opinion was initially based on observations that substantially higher doses of conventionally fractionated radiotherapy (RT) must be employed to achieve the same level of clinical response produced with lower dose for most other histologies [1]. For the case of melanoma, laboratory studies in the early 1970s suggested that higher radiation doses per fraction would be needed to achieve effective cell kill [2]. Subsequently, clinical investigations of hypofractionated RT were initiated to evaluate this approach to enhance radiation cytotoxicity [3].

Clinical outcomes reported in the 1980s tended to support the prevailing pessimistic viewpoints about RCC and melanoma response to RT. A dose-response relationship for palliative effect was observed by Onufrey and Mohiuddin among 125 patients treated for metastatic RCC [4], though their results were somewhat at variance with those of Halperin and Harisidias [5]. Multiple melanoma randomized studies were performed both in Europe and in the United States to explore ways to refine the use of RT in that setting: a Danish study found equivalence between 27 Gy in 3 fractions and 40 Gy in 5 fractions, and an RTOG study likewise found equivalence between 50 Gy in 20 fractions and 32 Gy in 4 fractions in terms of response rate [6,7].

More recently, high single doses of radiation delivered during stereotactic radiosurgery (SRS) to brain and spinal metastases have been studied in both melanoma and RCC, with encouraging outcomes [8-13]. Pre-clinical evidence has likewise indicated that a multi-session, high dose per fraction regimen of the type commonly used for stereotactic body radiation therapy (SBRT) is effective in the treatment of RCC [14], an observation further supported by clinical observations [15,16]. To our knowledge identical pre-clinical studies have not been reported for melanoma.

The increasingly popular use of high dose per fraction, SBRT-type regimens for not only melanoma and RCC but also for a variety of other lesions [17,18] has prompted a re-analysis of the traditional linear-quadratic (LQ) model-based formalism for predicting the radiation dose-response relationship for SBRT, since there is reason to consider that the LQ model overestimates radiation-induced cytotoxicity at high dose per fraction [19]. To begin to understand the potential benefits of SBRT for these histologies, we undertook a review of our institutional experience at the University of Colorado involving the use of SBRT for RCC and melanoma.

The first objective was to analyze whether the local control rates reported for high dose per fraction cranial and spinal SRS for RCC and melanoma can be replicated in other sites. Second, we attempted to model the SBRT dose-response relationship. In this context, we used both a traditional linear-quadratic model-based metric, the biological equivalent dose (BED), and a novel index proposed for modeling high dose per fraction RT, the single fraction equivalent dose (SFED)[19]. Finally, we reviewed the clinical observations typically seen in terms of metabolic imaging following SBRT for RCC and melanoma and the overall survival of this population of patients, with the intent of offering guidance for proper patient selection.

## Methods

We retrospectively reviewed all patients with melanoma and RCC treated with SBRT to metastatic sites from October 2004 to November 2009 at the University of Colorado. This study was approved by the University of Colorado Institutional Review Board. All patient charts were reviewed for clinical information including treatments with systemic therapies. Patients were excluded for review if they did not have any follow-up imaging after SBRT. Patients were considered to have oligometastatic disease if they had three or fewer sites of metastases in which all sites were treated with aggressive local therapy with possible systemic therapy. Otherwise, patients were classified as having extensive metastatic disease. Patients with extensive disease had relatively stable systemic disease with either painful lesions or growing lesions which were treated with SBRT.

SBRT was defined as a minimum total dose of 40 Gy given in 5 or fewer fractions using stereotactic technique previously described [20]. Briefly, for treatment planning, the gross tumor volume (GTV) was considered equal to the clinical target volume (CTV). The planning target volume (PTV) was typically constructed by adding 5 mm radially and 5-10 mm in the superior-inferior direction. The dose was prescribed to cover at least 95% of the PTV, normalized to the isodose line representing 60-80% of the maximum dose inside the PTV. The majority of plans were generated using multiple dynamic conformal arcs with at least 1 non-coplanar arc or a combination of multiple static beams. Localization was performed with KV orthogonal imaging fused to planning CT with the isocenter re-marked after shifts. Patients then underwent CT simulation for verification that the newly marked isocenter was within the GTV. In recent years, after the acquisition of 4D CT simulation technology, when significant breathing-related motion was present, the PTV was constructed by enlarging the internal target volume (ITV) defined on a 4D imaging set by 5 mm in all directions. Patients underwent abdominal compression to limit respiratory motion.

Toxicity was scored according to the Common Terminology Criteria for Adverse Events v3.0. The use of RECIST (Response Evaluation Criteria in Solid Tumors) criteria after SBRT is difficult in view of the expected parenchymal changes commonly seen in surrounding normal tissue within the volume that receives approximately 20 Gy or higher. For this reason, we did not characterize lesions as having had a complete response or partial response by RECIST criteria. Instead, local failure was scored when one of the following conditions were met: (1) tumor viability as seen by an increase in SUV on follow-up PET scan relative to the most recent prior PET; (2) expansion of a solid mass with discrete borders within the treated PTV by 20% in longest dimension relative to the most recent prior CT or MRI; or (3) tumor viability as evidenced pathologically by biopsy. In questionable cases, the follow-up CT was fused with the planning CT to define in-field LC. If a patient with suspicious failure was subsequently treated for that lesion with chemotherapy, the lesion was considered a failure. Overall survival (OS) was recorded from the date of treatment completion to last follow-up or date of death.

The SBRT dose regimen used was then converted to single fraction equivalent dose (SFED) using the following equation:

with D_q _estimated at 1.8 from the Park analysis [19]. Local control curves were generated using Kaplan-Meier method. Comparisons between curves were performed using the log rank method. Candidate predictors for local control (total dose, GTV, histology etc) were also evaluated by log rank analysis. Univariate analysis was performed with the median value using log rank comparisons (GraphPad Prism^®^, GraphPad Software, Inc., La Jolla California).

The dose-response relationship was modeled using a logistic tumor control probability (TCP) formula [21]:

Where D is the total dose, TCD_50 _is the dose that achieves 50% tumor control, and k describes the slope of the curve. Doses to individual lesions were grouped into tertile bins, and the x-axis value was the mean dose given in that bin, expressed as either BED or SFED, while the y-axis value was the probability of LC at twelve months.

In patients undergoing surveillance with PET scans who had long term local control, we looked at the pattern of the maximal standardized uptake value (SUV) change. Only patients with a pre-treatment and at least one post-treatment PET scan were included for analysis. The PET scans were performed intermittently for tumor surveillance and regularly in patients undergoing chemotherapy for other sites of disease. The lesions were contoured using dedicated medical image analysis software (MIMvista^®^, MIM Software, Inc., Cleveland, Ohio). This was then fused to their follow up PET scans and the maximum SUV (SUV_max_) was calculated for each lesion on each PET scan performed. Nine patients with 12 lesions had a total of 43 PET scans prior to and after SBRT.

## Results

### Patient population

Thirty patients with 53 treated lesions met the study inclusion criteria and were analyzed. Overall, 17 melanoma patients had 28 lesions, and 13 RCC patients had 25 lesions available for review. Two patients with RCC did not have follow-up imaging and were not included, one melanoma patient had an additional lesion that was treated but did not have any follow-up imaging and this lesion was excluded from our analysis. Patient ages ranged from 36 to 83, with median age of 59. There were 17 males and 13 females treated with SBRT. Seventeen patients had oligometastatic disease at time of treatment with all sites treated with SBRT, and 13 patients had extensive disease in which only selected lesions were treated with SBRT. The median number of lesions treated per patient was 2 (range, 1-3). Among the tumor sited treated, lung was most common (n = 39), followed by liver (n = 11) and bone (n = 3).

The SBRT regimens were 40-50 Gy delivered in 5 fractions (n = 23) or 42-60 Gy delivered in 3 fractions (n = 30). The regimen applied was selected at the discretion of the treating physician in view of clinical objectives and normal tissue dose considerations for each lesion without regard to the histology. The aim was to safely deliver the highest dose possible while respecting the surrounding normal tissue tolerance. The most common regimen was 60 Gy in 3 fractions (n = 20) followed by 45 Gy in 5 fractions (n = 11) and 50 Gy in 5 fractions (n = 8). Median gross tumor volume (GTV) was 6.3cc (range, 1-275). Median follow-up for patients alive at the time of analysis was 28.0 months (range, 4-68). See table 1 for treatment characteristics including SFED and BED values for each regimen.

**Table 1:** Treatment Characteristics

Fractionation Schedule	# of pts	SFED (Gy)	BED (Gy)
60 Gy in 3 fractions	20	56.4	180
54 Gy in 3	3	50.4	151.2
50 Gy in 5	8	42.8	100
45 Gy in 3	5	41.4	112.5
42 Gy in 3	2	38.4	100.8
45 Gy in 5	11	37.8	85.5
40 Gy in 5	4	32.8	72

Fractionation schedules and conversion to single fraction equivalent dose (SFED) and biological equivalent dose (BED).

### Tolerance and other therapies

There were no acute side effects, only mild late toxicities which were not dose dependent. Six patients experienced grade 1 toxicity (3 pain, 2 cough and 1 dyspnea). There was one incident of grade 3 toxicity of hypoxia at 11 months after treatment in an asthmatic patient who developed multiple pulmonary metastases requiring increased continuous oxygen use. One patient developed grade 3 radiation pneumonitis successfully managed with steroids.

Seven patients were treated with sorafenib, 5 before SBRT and 2 after SBRT as well as 7 patients treated with sunitinib. One patient underwent SBRT while sunitinib was held for 2 weeks before and after treatment, 3 patients were treated with sunitinib before SBRT and 3 patients were treated after SBRT. There was no significant increase in toxicity seen in these 14 patients (two grade 1 events and one late grade 3 pneumonitis). One patient with melanoma received CTLA4 antibody after radiation and did not experience any adverse side effects from SBRT. Overall patients were pre-treated with a variety of systemic therapies. The median number of courses was 1 with range 0-3. Additionally, patients went on to further systemic therapy with a median of one course (range 0-5).

### Local control and overall survival

**Figure 1:**
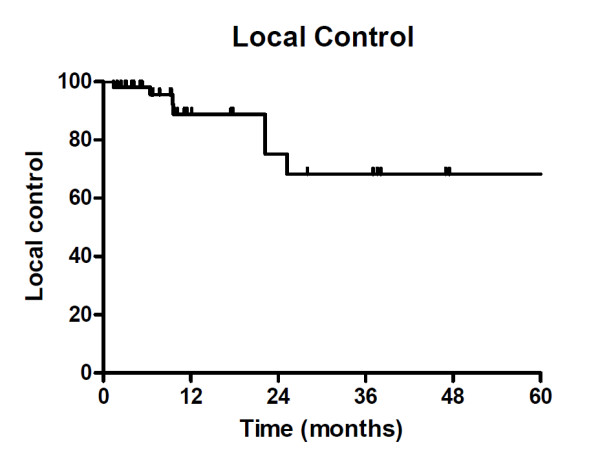
**Local Control**. Actuarial local control for both melanoma and RCC lesions

The actuarial rate of LC for all patients was 88% at 18 months (Figure 1). Several factors were analyzed by univariate analysis in an effort to identify predictors of LC. In general, for quantitative parameters, the median value was chosen as an arbitrary cut-off for univariate analysis to maximize the comparison cohorts. Log rank comparison revealed number of fractions (3 vs 5, p < 0.01) as well as dose per fraction (> 11 Gy/fraction vs <11 Gy/fractions, p < 0.01) and BED ( > 100 Gy vs < 100 Gy, p < 0.01) to be significant predictors of LC. Histology (RCC vs melanoma, p = 0.06) total dose (≥50Gy vs <50Gy, p = 0.09) SFED (≥ 45 Gy vs < 45 Gy, p = 0.06) and GTV (>7cc vs <7cc, p = 0.06) showed a strong trend towards significance. Site treated (lung vs other) and disease burden (oligometastatic vs widely metastatic) were not predictors of local control. Given the small number of events available to analyze, a multivariate analysis was not performed.

**Figure 2:**
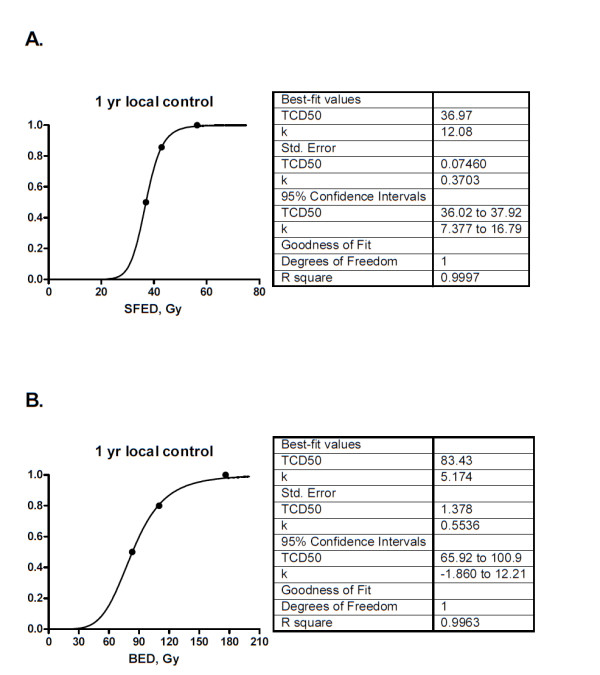
**Tumor Control Probability**. Tumor Control Probability graphs generated from dose response relationship modeling. Doses to individual lesions were grouped into tertile bins and the x-axis value was the mean dose given in that bin, expressed as either (a) SFED or (b) BED. The y-axis value was the probability of LC at 12 months.

We generated TCP graphs using both SFED and BED (Figure 2). Both SFED and BED had a strong coefficient of determination to predict future outcomes (SFED R = 0.999 and BED R = 0.996). Using the SFED TCP graph, a 90% chance of tumor control was calculated to an SFED of 44.3 Gy which translates into approximately 48 Gy in 3 fractions. Using BED, 90% chance of tumor control was calculated at 126 Gy, which corresponds to approximately 49 Gy in 3 fraction regimen.

**Figure 3:**
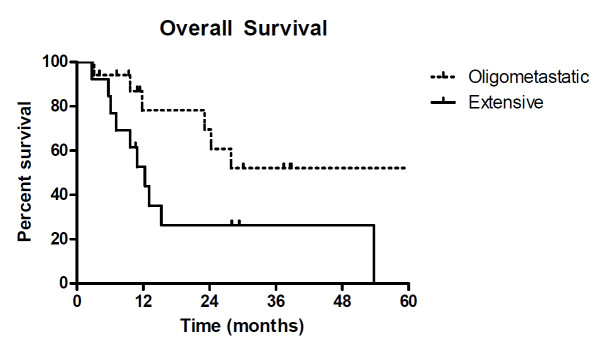
**Overall survival**. Actuarial overall survival of patients based on disease state. Oligometastatic disease was defined as three or less metastases in which all site of disease were treated with aggressive local therapy. Extensive disease was defined as patients with more than three sites of metastases.

Median overall survival for all patients in this study was 24.3 months. The median overall survival of patients with oligometastatic disease was not reached while patients with extensive metastatic disease had a median overall survival of only 12.3 months (p = 0.03) (Figure 3). Median overall survival was not reached in patients with RCC, and was statistically longer than melanoma patients with median overall survival of 22.2 months (p = 0.015).

### Metabolic imaging and kinetics of PET scan changes

**Figure 4:**
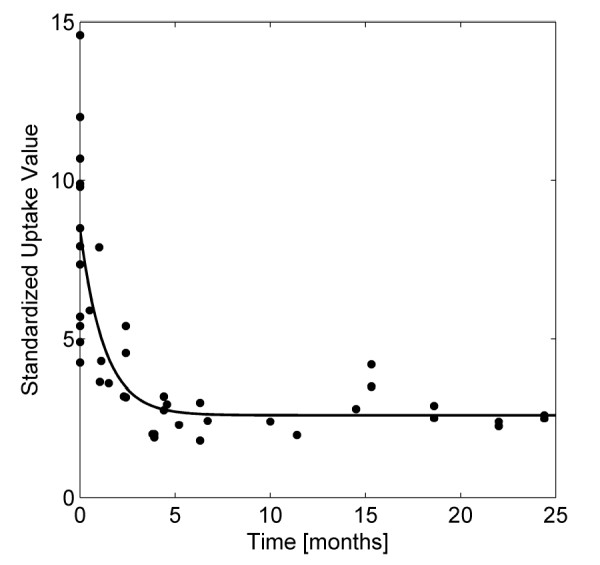
**Change in SUV for controlled lesions**. The Standardized uptake values were plotted with pre-treatment PET used for planning as time 0. Follow-up PET/CT's were fused and SUV was generated for each treated lesion that was controlled. An exponential equation was generated revealing a post-treatment baseline level of activity of 2.6 at 7 months.

The SUV_max _was plotted and fitted with an exponential equation. The median pre-treatment SUV_max _was 7.9 (range 1.5 - 14.6). The calculated time for the SUV_max _value to decrease by half the original value was 3.8 months (Figure 4). We found that the calculated post-treatment baseline SUV_max _was 2.6, which was reached at approximately 7 months. The median post-treatment SUV_max _was 2.5 (range 1.8 - 3.2).

## Discussion

We have observed in a cohort of patients treated with SBRT for metastatic melanoma or RCC, a high rate of durable LC can be achieved, especially for patients with a 3 fraction SBRT total prescription dose on the order of 48-49 Gy or higher. It should be appreciated that this dose estimate represents the dose covering the periphery of the PTV and that substantial dose hotspots are always created in the GTV. Thus, the actual dose need to ablate the gross disease itself is higher than this estimate.

The data were evaluated in terms of SFED and BED because these indices incorporate both the total dose delivered as well as the dose per fraction. SFED was designed to analyze the effect of high dose per fraction exposure by using an equation for cell survival which, when plotted a logarithmic scale, initially curves downward with increasing dose in a similar way as an LQ-based curve but then straightens at higher doses, correcting for an overestimation of cell kill by BED in the SBRT/ablative dose range [22]. There are at least two reasons why the BED might not characterize high dose effects as well as a model such as the SFED. First of all, there is the phenomenon recognized long ago whereby for lengthy individual exposures of living cells to radiation, intra-exposure repair can occur, obliging a correction to the simple LQ model that adjusts for this process. This notion was advanced at least as long ago as the 1940s, when Lea and Catcheside modeled radiation-induced chromosomal aberrations in a plant model using a linear-quadratic formula that also could be modified with a factor that accounted for the total time of exposure [23].

A second, more modern explanation of why BED might not precisely model high dose effects relates to a mechanism of tumor cell kill at work in vivo that is not active in vitro. With conventionally fractionated doses, radiation cell kill is assumed to be largely mediated through oxygen dependent DNA damage with resulting loss of clonogenicity, an effect seen in vitro and presumed to occur in vivo. However, pre-clinical studies have suggested that the high doses of radiation delivered in each session of SBRT might trigger an entirely different method of cell kill in vivo via an anti-angiogenic pathway involving endothelial cell apoptosis [24]. Coincidentally, apropos of the present clinical series, the pre-clinical studies initially suggesting this mechanism included studies of melanoma xenografts. Furthermore, endothelial cell apoptosis appeared to be induced above a threshold dose of 11 Gy, and the present study similarly suggested significant improvement in tumor cell kill with a fraction size above that level. Of course, melanoma and RCC have also been shown to have a large initial shoulder on the cell survival curve [25], and the present study's favorable results might also be at least partly explained by the fact that doses in the SBRT range exceed that of the initial shoulder region. Both BED and SFED proved to be a reliable predictor for LC. Further studies will be needed to resolve whether one is truly superior to the other, and it will be informative to see the results of RTOG 0915 in which a single 34 Gy fraction is compared to 48 Gy in 4 fraction regimen for primary lung cancer. The SFED model would predict better LC with the 48 Gy arm, while BED modeling predicts the single 34 Gy treatment to have superior LC.

The present clinical observations of high LC after aggressive radiation treatment are consistent with what has been observed after single high dose SRS to brain and spinal metastases for both melanoma and RCC [8-10,26]. In these studies the LC for melanoma is typically lower than for RCC [10,26], for which brain SRS can achieve very high LC [11]. Likewise, in the present study we observed a trend for lower 1 year LC for melanoma than RCC (82% v. 95%), possibly intrinsic differences in radiosensitivity that are retained even in the high dose-per-fraction setting. In a study of SBRT in primary and metastatic RCC, the local control rate was 90-98% [16] which is in line with our own and other institutional local control rates across a broad range of histologic subtypes [16-18,27,28].

The oligometastatic hypothesis suggests that tumors early in systemic disease progression may present with a limited number of discrete lesions without extensive occult spread of disease, thus a condition amenable to potentially curative intervention if the identifiable lesions can be eradicated [29]. Studies of liver metastectomy in patients with RCC reveal that there are long term survivors and chance for cure with a 5 year OS rate of 39% [30]. The argument for using ablative local therapy for isolated metastases is strengthened if effective systemic therapy is available to complement it [31]. And in recent years, for both melanoma and RCC there have been new systemic agents developed that provide clinical benefit, including the anti-CTLA-4 antibody, ipilimumab, and multi-targeted agents such as sunitinib and sorafenib.

Properly selected patients with metastatic RCC undergoing lung resection have a chance for long term survivorship [32], as do patients with liver metastases from RCC, where a 5 year OS of approximately 40% has been reported for a group of well selected patients [30]. Patients with liver metastases from RCC tend to fare better than patients with liver metastases from melanoma [33], once again suggesting basic differences in the typical degree of aggressiveness between these cancer types. In the present series, melanoma patients likewise had shorter median survival than RCC patients.

In this series the arbitrary cutoff point applied to characterize patients as having oligometastatic vs. extensive metastatic disease was the presence of 3 or fewer individual sites of disease. The superior outcome of patients defined as oligometastatic by this definition was expected, and this or a similar cutoff level of sites of disease would appear to be appropriate as a stratification variable for future studies of SBRT in the treatment of metastatic disease. However, the difference in outcome between the cohorts defined in this manner does not rule out the possibility that patients with more extensive disease might still benefit from a general reduction in their systemic disease burden, whether achieved by systemic therapy or local therapy. Indeed, for the case of RCC in particular, two independent phase III studies indicate that a reduction in a patient's total burden of disease via nephrectomy lengthens OS for patients with known metastatic disease, even though not all sites of disease were locally treated [34]. Thus, as studies are designed in the future, it is important to avoid the overly narrow assumption that only patients with oligometastatic disease can potentially benefit from ablation of metastatic sites of disease via local therapy, though certainly patients with more limited disease will have a better prognosis overall.

PET scans are now widely available to monitor response to cancer therapy in a variety of setting, and we have here reported on the kinetics of change in metabolic activity following SBRT for RCC and melanoma. In our cohort of locally controlled patients, the decrease to a steady post-treatment baseline SUV_max _took approximately 7 months. The post-treatment baseline level averaged 2.6 and was consistent with findings of Henderson et al, who showed that almost half of primary non-small cell lung cancer lesions have moderately elevated SUV_max _at 12 months without local failure [35]. Hoopes also reviewed follow-up PET scans in patients undergoing SBRT for NSCLC and found that 14% of patients had moderate hypermetabolic activity without local failure 20 months after SBRT completion [36]. In addition to the baseline level activity, we found the average time to decrease the post-treatment SUV_max _by half the value took 3.8 months. The residual activity observed after treatment likely represents energy-dependent inflammatory and tissue-reparative responses, but further analysis of the nature of the lingering metabolic activity is beyond the scope of the present study.

The present study results are the first to support independently the observations of Wersall and colleagues [16], who likewise saw longer survival in RCC patients with oligometastatic disease compared with more extensive disease. Furthermore, we here have analyzed data using the recently proposed SFED metric, which at least in this relatively small experience proved a robust predictor for LC. The present study likewise generates the testable hypothesis that with adequately aggressive non-invasive SBRT regimens incorporating high dose per fraction schedules, the rates of LC achieved even for classically "radioresistant" histologies appear similar to what can be achieved for histologic subtypes expected to be more radiosensitive.

## Conclusions

The present study demonstrates that an aggressive SBRT regimen is an effective modality for controlling metastatic melanoma and RCC. The LC rates achieved in our series are comparable to those obtained with SBRT for other tumor histologies, suggesting a dominant mechanism of in vivo tumor ablation after high dose fractions that largely overrides intrinsic differences in cellular radiosensitivity between histologic subtypes of tumor. SFED TCP modeling indicates that to achieve a high rate of durable LC in a 3 fraction regimen of SBRT, a dose of at least 48 Gy is required.

## Competing interests

The authors declare that they have no competing interests.

## Authors' contributions

MAS conceived of the study, carried out data collection, performed a literature search, and drafted the manuscript. BK participated in the design, literature research, statistical analysis, and drafting the manuscript. TES participated in study design and data retrieval. RG, KL, WR, MG, and MC contributed to the clinical management of patients and data collection. TF contributed to patient management and in drafting the manuscript. DR participated in the design, clinical patient management, and manuscript writing. All authors read and approved the final manuscript.
